# NIDS-FGPA: A federated learning network intrusion detection algorithm based on secure aggregation of gradient similarity models

**DOI:** 10.1371/journal.pone.0308639

**Published:** 2024-10-24

**Authors:** JiaMing Wang, Kai Yang, MinJing Li

**Affiliations:** 1 Xi’an Key Laboratory of Human-Machine Integration and Control Technology for Intelligent Rehabilitation, School of Computer Science, Xijing University, Xi’an, P.R. China; 2 School of Computer, Xijing University, Xi’an, China; Jinan University, CHINA

## Abstract

With the rapid development of Industrial Internet of Things (IIoT), network security issues have become increasingly severe, making intrusion detection one of the key technologies for ensuring IIoT security. However, existing intrusion detection systems face challenges such as incomplete data features, missing labels, parameter leakage, and high communication overhead. To address these challenges, this paper proposes a federated learning-based intrusion detection algorithm (NIDS-FGPA) that utilizes gradient similarity model aggregation. This algorithm leverages a federated learning architecture and combines it with Paillier homomorphic encryption technology to ensure the security of the training process. Additionally, the paper introduces the Gradient Similarity Model Aggregation (GSA) algorithm, which dynamically selects and weights updates from different models to reduce communication overhead. Finally, the paper designs a deep learning model based on two-dimensional convolutional neural networks and bidirectional gated recurrent units (2DCNN-BIGRU) to handle incomplete data features and missing labels in network traffic data. Experimental validation on the Edge-IIoTset and CIC IoT 2023 datasets achieves accuracies of 94.5% and 99.2%, respectively. The results demonstrate that the NIDS-FGPA model possesses the ability to identify and capture complex network attacks, significantly enhancing the overall security of the network.

## 1 Introduction

Intrusion detection is a pivotal aspect within the network security defense framework, aiding cybersecurity personnel in the timely identification and interception of malicious activities, thereby safeguarding network privacy and information integrity. In the current intricate and ever-evolving landscape of cyber threats, traditional security defenses alone prove inadequate in combatting the myriad of complex attack vectors. Hence, the research and advancement in intrusion detection emerge as imperative.

Currently, most deep learning-based network intrusion detection models are built under the condition of having sufficient network attack data. However, in practical applications, individual organizations can only rely on their own data to train and analyze these models. These datasets typically contain few high-quality, well-labeled attack samples. Consequently, models trained on such limited data often suffer from low detection rates, high false alarm rates, and poor generalization ability.

An effective solution to these problems is to upload multi-party data samples to high-computing platforms and centrally utilize these data to train models. However, data from multiple data owners often contain sensitive internal information, which results in data owners being reluctant to share their data. Therefore, addressing the issues of insufficient data and protecting sensitive information becomes crucial in network intrusion detection. Introducing Federated Learning (FL) into intrusion detection is an effective approach to tackle these challenges.

Bukhari et al [1]. proposed a novel intrusion detection method based on a stacked convolutional neural network and bidirectional long-short-term memory (SCNN-Bi-LSTM) model. This method utilizes FL mechanisms to enhance the performance of intrusion detection systems (IDS) in wireless sensor networks (WSN) while protecting data privacy. Through a unique FL design, it effectively identifies complex network threats, significantly improves IDS performance, and highlights the potential of FL and deep learning in enhancing WSN security and privacy. However, the method encounters challenges in handling non-iid data, leading to difficulties in model convergence and potential increases in communication overhead. Additionally, the model’s robustness in the presence of malicious users still needs improvement. However, the method encounters difficulties in model convergence when handling Non-IID(Non-Independent and Identically Distributed) data, which may lead to increased communication overhead. Additionally, the model’s robustness against malicious users still requires improvement.

Rashid et al [2]. proposed a federated learning-based approach aimed at safeguarding the security and privacy of Internet of Things (IoT) networks. By collectively training local IoT device data and devising a parameter update filtering and aggregation method, they reduced the impact of local models on the global model while enhancing the overall model performance. This method achieved an accuracy of 92.49% on the Edge-IIoTset dataset, which is close to the accuracy of traditional centralized machine learning (ML) models (93.92%). However, the method lacks effective weight allocation for the contribution of local models during the aggregation phase, which still results in communication overhead and convergence issues. Additionally, although its accuracy on the Edge-IIoTset dataset is comparable to traditional centralized machine learning models, its detection performance needs further validation in more complex and dynamic network environments.

Sarhan et al [3]. proposed a collaborative network threat intelligence sharing scheme that enables multiple organizations to collectively design, train, and evaluate ML-based network intrusion detection systems. By utilizing the NF-UNSW-NB15-v2 and NF-BoT-IoT-v2 datasets for design and evaluation, they employed FL mechanisms to avoid sharing sensitive user information, thereby protecting data privacy. Additionally, they designed and evaluated a universal ML model capable of effectively classifying various benign and intrusive traffic types from multiple organizations without the need for inter-organizational data exchange. However, when dealing with highly heterogeneous network traffic, the performance of its general ML model still requires improvement, especially when facing significant differences in data characteristics across different organizations. The model’s generalizability and accuracy may be affected.

Lai et al [4]. proposed a two-stage defense mechanism called Defending Poisoning Attacks in Federated Learning (DPA-FL), specifically designed to address poisoning attacks in FL, particularly applicable in the field of intrusion detection. The first stage utilizes relative differences for rapid comparison of weights between participants to distinguish between malicious and benign local models. The second stage involves testing the accuracy of the aggregated model and identifying attackers when there is a decrease in accuracy. Experimental results demonstrate that DPA-FL achieves a 96.5% accuracy in defending against poisoning attacks. Compared to other defense mechanisms, DPA-FL can improve F1 scores by 20% to 64% under backdoor attacks. Additionally, when the number of attackers is small, DPA-FL can exclude attackers within twelve rounds. Although DPA-FL performs excellently in defending against poisoning attacks, its accuracy and F1 score still need optimization when dealing with a limited number of attack categories. Additionally, the method has a potential for misjudgment when distinguishing between malicious and benign participants, which may impact the accuracy and robustness of the global model.

Doriguzzi-Corin et al [5]. introduced an adaptive FL method named Federated Learning Approach to DDoS (FLAD) for DDoS attack detection in network security applications. This method addresses the convergence issue of FL processes in dynamic network security scenarios, achieving frequent model updates for the latest attack features without the need to share testing data to monitor the performance of the trained model. By employing the latest DDoS attack datasets, studies indicate that FLAD outperforms existing FL algorithms in terms of convergence time and accuracy, demonstrating good robustness on unbalanced heterogeneous DDoS attack datasets. However, when dealing with highly heterogeneous and dynamically changing network attack characteristics, the detection capability and model update frequency of the FLAD method still need further enhancement to ensure its effectiveness and robustness across different attack scenarios.

Sáez-de-Cámara et al [6]. conducted research on an unsupervised model training architecture for network intrusion detection in large-scale distributed IoT and IIoT deployments. They utilized FL to enable collaborative training among peers, reducing data isolation and network overhead issues. Additionally, they fully integrated an unsupervised device clustering algorithm into the FL pipeline to address heterogeneity issues arising in FL environments. Through implementation and evaluation on a testbed containing various simulated IoT/IIoT devices and attackers, they evaluated the anomaly detection model and demonstrated its effectiveness in real-attack scenarios. However, the unsupervised model training framework still faces challenges in training and optimization within the FL environment, and its effectiveness and stability in real-world attack scenarios require further validation.

In summary, in practical scenarios, data among different clients often exhibits the characteristic of Non-IID. This feature leads to significant differences in data distribution, thereby resulting in uneven quality of local models. During the model aggregation phase, some local models, due to their minor contributions, may even have a negative impact. This not only increases communication rounds but also may lead to difficulties in model convergence, significantly increasing the overhead of network interaction. More seriously, there may be malicious users within the client group who train local models by injecting extreme data. These models differ significantly from the global model update, thereby weakening the performance of the global model and exacerbating the overhead burden between clients and servers. To effectively address the above issues, this paper designs a gradient-similarity-based model aggregation algorithm targeting these problems specifically. By setting model filtering and weighting, the algorithm resolves the aforementioned issues.

Unlike the aforementioned research, we first transform network traffic data into grayscale images and combine a deep model consisting of 2D-CNN and BIGRU to process these grayscale image data. This approach overcomes the limitations of traditional deep learning models in handling missing data features and enhances the model’s ability to detect complex network attacks. During the model aggregation process, we consider the similarity between each local model update and the global model update, calculating the contribution of each local model to the global model. Consequently, we design methods for model filtering and dynamic weighted aggregation to mitigate the impact of low-quality and low-contribution local models on the global model, thereby reducing communication overhead between participants and the central server. Additionally, we address security issues in the federated learning process by introducing Pallier homomorphic encryption technology to ensure the secure transmission of model parameters. Ultimately, our model achieves faster convergence speeds and higher detection performance on Non-IID clients. In summary, our research provides a novel solution for improving network intrusion detection effectiveness and safeguarding data security.

The main contributions of this study are as follows:

1. We converted the traffic data of industrial IIoT into grayscale image data and employed a deep model combining 2D-CNN and BIGRU to process this data. This approach overcomes the limitations of traditional deep learning models in handling missing data features when dealing with network traffic data, thereby enhancing the recognition and capture capabilities of complex network attacks.
2. We propose the GSA algorithm. This algorithm screens the clients participating in FL. In the model aggregation stage, different weights are assigned to local models based on the contribution of clients to achieve dynamic weighting, with the aim of reducing communication rounds, achieving rapid model convergence, and decreasing communication overhead.
3. This study effectively addresses the issue of potential inference of source data by attackers during the training process by introducing homomorphic encryption technology. We utilize public and private keys generated by a trusted third-party entity to encrypt gradient update parameters locally on the client-side, which are then uploaded to a central server. The central server employs the private key to decrypt and aggregate these encrypted parameters, thereby ensuring the privacy and security of the model parameters.

The structure of this paper is as follows: Section 1 introduces the background, significance, contributions, and current relevant work of this research. Section 2 elaborates on the fundamental knowledge, including FL, CNN, BiGRU, Gradient Similarity Model Aggregation Algorithm, and Paillier Homomorphic Encryption Algorithm. Section 3 discusses the establishment process of the NIDS-FGPA model, providing detailed explanations of the implementation methods of its key components and the handling of experimental datasets. Section 4 describes the construction of the experimental environment, including the definition of the environment and parameters, as well as the evaluation metrics used in the experiments. Section 5 presents the results of simulation experiments, analyzing the comparison between the GSA and previous algorithms, as well as the impact of parameters in the Paillier Homomorphic Encryption Algorithm, and determining the optimal parameters. The performance and effectiveness of NIDS-FGPA are analyzed through multi-classification experiments, demonstrating its advantages through comparative experiments. Finally, in Section 6, we summarize the entire content and propose prospects for future work.

## 2 Elaboration of fundamental knowledge

### 2.1 The basics knowledge of federated learning

Traditional ML and deep learning methods typically assume a centralized environment, where data, when abundant, is stored centrally on a cloud service platform with high computing capabilities for model training. However, in real-world scenarios, data owners are often unwilling or unable to share their data due to reasons such as privacy concerns, business competition, and technological barriers. This reluctance stems from the potential presence of sensitive information in the data, which, if shared, could lead to serious issues such as data breaches [7]. If model training relies solely on proprietary data, the model may become trapped in local optima due to insufficient data and may fail to capture the characteristics of other data owners, thereby diminishing its generalization ability and robustness.

**Fig 1:**
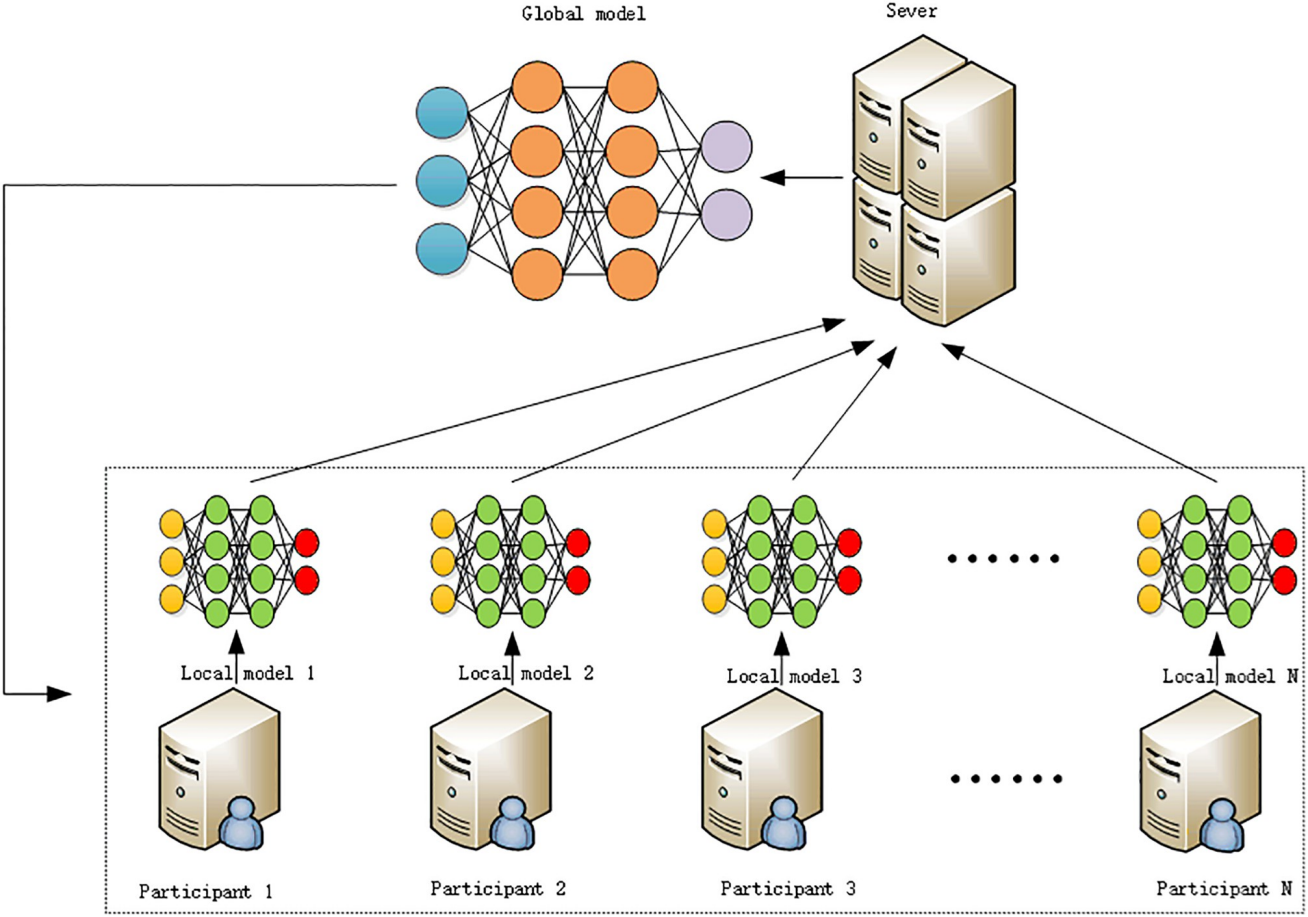
Federated learning framework diagram.

To address the aforementioned issues, FL has emerged. On the one hand, this method relocates the model training process from a centralized cloud platform to local devices possessing data, dispersing the computational load and enabling data training locally. On the other hand, by integrating previously inaccessible data, FL allows the model to learn from multi-party data, thus enhancing its generalization ability and robustness. Therefore, FL not only addresses the problem of data scarcity but also effectively protects data privacy. Its framework model, as depicted in Fig 1, mainly consists of clients and a central server, with the cloud service platform regarded as the central server and data owners serving as clients participating in the learning process [8].

The central server is primarily responsible for coordinating the global learning objective. Multiple distributed clients (such as mobile phones, IoT devices, etc.) use their own local data to train local learning models separately. Eventually, the server aggregates the trained models into a global model [2]. Training is an iterative process that involves local model updates and global model aggregation, repeating until the model converges or reaches a predetermined number of iterations.

Let’s define *C* clients 1, 2, 3, …, *C* with their respective datasets *D*_1_, *D*_2_, *D*_3_, …, *D*_*C*_. The communication round index is *r*, and the client index is *c*. We set the global model as *G*, the local model as *L*, the global model parameters as *W*, and the local model parameters as *w*. The process of one communication round is outlined below:

1. Participating clients download the global model *G*^*r*−1^ from the server.
2. Each client receives *G*^*r*−1^ and trains its local model $L_{c}^{r}$ using local data.
3. The clients then upload the model parameters or gradient updates of $L_{c}^{r}$ to the central server.
4. After receiving the model parameters or gradient update data from each party $L_{c}^{r}$, the server performs an aggregation operation to derive global parameters or global gradient updates, thereby constructing the global model *G*^*r*^. Here, $L_{c}^{r}$ represents the local model of the *c*-th client during the *r*-th round of communication, while *G*^*r*^ denotes the global model for the *r*-th round of communication.
5. If the global model *G*attains convergence or completes the stipulated number of communication rounds, the communication process shall be terminated. The core objective of federated learning is to minimize the global loss through ongoing communication, training, and updates, ultimately achieving convergence of the model:
  $$\begin{array}{c} \underset{W}{\text{min}}\;f\left(W\right)=\sum_{c=1}^{C}\varphi_{c}\cdot f_{c}\left(W\right) \end{array}$$ (1)
  *f* represents the global loss, *f*_*c*_ represents the local loss, and *φ*_*c*_ is the weight of the local model during aggregation.

#### 2.1.1 The categorization of federated learning

**Fig 2:**
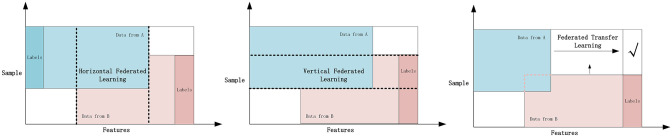
Federated learning classification.

Based on different data partitioning methods, federated learning can be divided into horizontal federated learning, vertical federated learning, and federated transfer learning, which are suitable for solving different practical problems. Specific classification descriptions are shown in Fig 2.

**Horizontal federated learning** is suitable for data distribution scenarios where datasets have similar feature sets but different samples. In this case, different datasets overlap in the feature space, but each dataset contains different samples (i.e., data points). By aggregating similar feature information from different data sources, horizontal federated learning can enhance model performance while protecting privacy [9].

**Vertical federated learning**, on the other hand, is applicable to scenarios where datasets have different feature sets but overlapping samples. In this mode, different datasets possess distinct feature sets but share the same set of samples. By integrating complementary feature information from various data sources, vertical federated learning can build more comprehensive and accurate models [10].

**Federated transfer learning** is a method that combines the ideas of transfer learning and federated learning, suitable for scenarios where there are significant differences in data distribution between the source domain and the target domain. In such cases, the source and target domains may differ both in the feature space and in the sample space. Federated transfer learning utilizes the rich knowledge from the source domain to assist learning in the target domain while maintaining data privacy and security. This is particularly useful in many cross-domain and cross-organizational scenarios for knowledge sharing and transfer [11].

### 2.2 Convolutional neural network

CNN is a powerful deep learning model, with its core lying in automatically extracting local features of images through convolutional layers, such as edges and textures. These features are then downsampled through pooling layers to reduce computational complexity and enhance feature robustness. Finally, these features are integrated through fully connected layers to output the final classification or regression results [12]. CNN’s advantages include its local perception and weight sharing properties, making it efficient and accurate in handling large-scale image data.

1D Convolutional Neural Network (1D-CNN) is primarily used for processing sequential data such as text and time series. Its convolutional operation is performed in only one direction and is typically used to extract local features from sequential data [13].

2D Convolutional Neural Network (2D-CNN) is widely used in the field of image processing. It can perform convolution operations in two dimensions of the image and is commonly used to extract spatial features from images, such as edges and textures [14].

3D Convolutional Neural Network (3D-CNN) is employed for handling data with a time dimension, such as video data. In addition to performing convolution operations in the spatial dimension, 3D-CNN can also conduct convolutions in the temporal dimension, thereby capturing spatiotemporal features in videos [15].

In mathematics, the specific operation of the convolutional layer in a CNN is detailed in Formula 2.

$$\begin{array}{c} H_{i,j}^{l}=f(\sum_{m=0}^{M-1}\sum_{n=0}^{N-1}W_{m,n}^{l}\cdot X_{i+m,j+n}^{l-1}+b^{l}) \end{array}$$ (2)

Where:

-
$H_{i,j}^{l}$ represents the activation at position (*i*, *j*) in the *l*-th layer,
- *f* denotes the activation function,
-
$W_{m,n}^{l}$ are the learnable weights (filters) of the *l*-th layer,
-
$X_{i+m,j+n}^{l-1}$ denotes the inputs from the previous layer,
- *b*^*l*^ represents the bias term.

In this formula, the convolution operation is applied to the input *X*^*l*−1^ with the learnable filters *W*^*l*^, followed by an element-wise activation function *f* and an optional bias term *b*^*l*^.

### 2.3 Bidirectional gated recurrent unit

BIGRU is a variant of recurrent neural networks (RNN), particularly suitable for processing sequential data such as text or time series. Its key lies in controlling the flow of information through gate mechanisms, effectively alleviating the vanishing gradient problem in traditional RNN. Additionally, the bidirectional structure of BIGRU enables it to simultaneously consider both forward and backward information of sequences, thus providing a more comprehensive understanding of the sequence context [16]. This makes BIGRU excel in tasks such as natural language processing and speech recognition.

Mathematically, the operation of a BIGRU unit can be represented as follows:

$$\begin{array}{c} z_{t}=\sigma (W_{z}\cdot \left[h_{t-1},x_{t}\right]+b_{z}) \end{array}$$ (3)

$$\begin{array}{c} r_{t}=\sigma (W_{r}\cdot \left[h_{t-1},x_{t}\right]+b_{r}) \end{array}$$ (4)

$$\begin{array}{c} {\tilde{h}}_{t}=\text{tanh}(W_{h}\cdot \left[r_{t}⊙h_{t-1},x_{t}\right]+b_{h}) \end{array}$$ (5)

$$\begin{array}{c} h_{t}=\left(1-z_{t}\right)⊙h_{t-1}+z_{t}⊙{\tilde{h}}_{t} \end{array}$$ (6)

Where:

- *x*_*t*_ is the input at time step *t*,
- *h*_*t*−1_ is the hidden state at time step *t* − 1,
- *z*_*t*_ is the update gate, controlling how much of the past information should be retained,
- *r*_*t*_ is the reset gate, controlling how much of the past information should be forgotten,
-
${\tilde{h}}_{t}$ is the candidate hidden state, representing the new candidate information,
- *W*_*z*_, *W*_*r*_, and *W*_*h*_ are the weight matrices,
- *b*_*z*_, *b*_*r*_, and *b*_*h*_ are the bias vectors, and
- *σ* is the sigmoid activation function.

In this formulation, [*h*_*t*−1_, *x*_*t*_] represents the concatenation of the previous hidden state and the current input, and ⊙ denotes element-wise multiplication [17].

### 2.4 Model aggregation algorithm for gradient similarity

In FL, numerous client-side local models generate updates after training, which are then aggregated to collectively guide the optimization direction of the global model. Whether a local update is useful in model aggregation optimization cannot be simply measured by the size of local data, as in the Federated Averaging (FedAvg) algorithm [18].

Considering the Non-IID nature of client data in reality, the update directions of some local models may significantly differ from those of other models or the global model. These updates with substantial differences not only contribute limitedly to the global update but may even have adverse effects, while also increasing communication overhead due to parameter transmission. Therefore, simply weighting by local data volume may lead to difficulties in global model convergence, poor performance, and increased communication costs [19].

To address this issue, this paper introduces similarity calculation to quantify the contribution of local updates to the global model. During the training process, each round initializes local model parameters *w*^*r*−1^ with the global model parameters from the previous round. Subsequently, the local model trains new parameters *w*^*r*^ based on these initial parameters through gradient descent [20]. The relationship between model gradients and model parameters can be expressed as follows:

$$\begin{array}{c} w^{r}=w^{r-1}-\rho \times ▽g^{r} \end{array}$$ (7)

In this formula, ▽*g*^*r*^ represents the gradient update, while *ρ* denotes the model’s learning rate. This formula is applicable not only for computing gradient updates on individual devices (or locally) but also, after aggregating local updates into global updates, for calculating the global model parameters of the current round based on the global model parameters from the previous round [21]. Thus, obtaining the latest global model.

Gradient updates exist in vector form, and in this paper, the cosine similarity between gradient update vectors is utilized to compute the similarity between local updates and global updates. suppose there are two vectors $\vec{a}$ and $\vec{b}$. If the angle between these two vectors is acute, indicating a sharp angle, it suggests that their directions are relatively close, implying a high degree of similarity, with a larger cosine similarity value. If the angle is a right angle, the directions are dissimilar [22]. If the angle is obtuse, the directions are opposite, indicating dissimilarity, with a smaller cosine similarity value.

In this paper, local updates and global updates are also analogized to this scenario, and the cosine similarity between them is calculated, as shown in Eq (8). Furthermore, the inverse cosine operation of cosine similarity is performed, as shown in Eq (9), to compute the angle between gradient update vectors [23]. This serves as the criterion for evaluating the similarity between local and global models in terms of model selection.

$$\begin{array}{c} s_{c}^{r}=\frac{▽g_{c}^{r}▽g^{r-1}}{∥▽g_{c}^{r}∥\times ∥▽g^{r-1}∥} \end{array}$$ (8)

$$\begin{array}{c} \begin{array}{cc} \theta_{c}^{r} & =\text{arccos}(s_{c}^{r})\\ & =\text{arccos}(\frac{▽g_{c}^{r}\cdot ▽g^{r-1}}{∥▽g_{c}^{r}∥\times ∥▽g^{r-1}∥}) \end{array} \end{array}$$ (9)

Where $s_{c}^{r}$ represents the cosine similarity between local gradient update $▽g_{c}^{r}$ and global gradient update $▽g^{r-1}$, and $\theta_{c}^{r}$ denotes the angle between the gradient vectors. If the angle is acute, i.e., $\theta_{c}^{r}<\pi /2$, the global gradient and the local gradient have similar directions, leading to a positive contribution during model aggregation. If the angle is large, i.e., $\theta_{c}^{r}>\pi /2$, indicating a right or obtuse angle, the global gradient and the local gradient have opposite directions, resulting in no contribution or a negative contribution during model aggregation [24].

### 2.5 Description of Paillier homomorphic encryption

Homomorphic encryption is an encryption technique that allows users to perform addition, subtraction, multiplication, and division operations directly on ciphertext and obtain the same output as those operations performed on plaintext. Its main advantage is that it can perform computations on encrypted data without compromising its confidentiality or integrity. Specifically, for given two pieces of plaintext data *a*_1_ and *a*_2_, the ciphertexts obtained after applying homomorphic encryption operations on them are represented as *En*(*a*_1_) and *En*(*a*_2_) [25]. This algorithm typically exhibits two characteristics: additive homomorphism and multiplicative homomorphism, specifically manifesting as follows:

Additive homorphism:

$$\begin{array}{c} En\left(a_{1}\right)⊕En\left(a_{2}\right)=En\left(a_{1}⊕a_{2}\right) \end{array}$$ (10)

Multiplicative homorphism:

$$\begin{array}{c} En\left(a_{1}\right)⊗En\left(a_{2}\right)=En\left(a_{1}⊗a_{2}\right) \end{array}$$ (11)

Currently, homomorphic encryption can be divided into two main types: partially homomorphic encryption and fully homomorphic encryption. Partially homomorphic encryption only allows simple or limited addition and multiplication operations to be performed on ciphertexts, such as addition and multiplication. In contrast, fully homomorphic encryption enables arbitrary computational operations to be performed on ciphertexts [26]. In the application scenarios of homomorphic encryption, there is typically a trusted third party responsible for processing data to ensure no leakage of any information. Commonly used homomorphic encryption algorithms include Paillier encryption, RSA encryption, and CKKS encryption. Table 1 provides a comparison of the security and computational complexity of the currently popular homomorphic encryption algorithms.

**Table 1:** Comparison of homomorphic encryption algorithms.

Homomorphic Encryption Algorithm	Security	Computational Complexity	Homomorphism
Paillier	High	Low	Additive, Multiplicative
RSA	Low	Low	Multiplicative
CKKS	High	High	Additive, Multiplicative (Approximate)
Gentry	High	High	Additive, Multiplicative

Taking into account security, computational complexity, and homomorphic properties, the Paillier algorithm is highly favored due to its relatively low computational complexity and high security. Furthermore, its support for both additive and multiplicative homomorphic operations makes it an ideal choice for enhancing security in our context [27]. Therefore, we have decided to introduce the Paillier algorithm into the industrial IoT intrusion detection algorithm based on federated secure aggregation, aiming to strengthen the security of the model.

The Paillier algorithm is a probabilistic public-key encryption algorithm that satisfies additive homorphism and achieves encryption by addressing problems based on composite residues. Currently, the Paillier algorithm has been widely applied in the fields of encrypted signal processing and third-party data processing [28]. Below is a brief description of the Paillier algorithm scheme:

(1) Key Generation
**Step 1:** Randomly choose two numbers *p* and *q* (both primes), ensuring that the greatest common divisor of *pq*, *p* − 1, and *q* − 1 is 1, and also ensuring that the lengths of *p* and *q* are equal.
**Step 2:** Calculate the value of *pq* and assign the result to *n*, then compute the least common multiple λ of *pq*, *p* − 1, and *q* − 1.
**Step 3:** Randomly select a positive integer *g*, ensuring that it is less than *n*^2^.
**Step 4:** Define a *L* function:
  $$\begin{array}{c} L\left(x\right)=\frac{x-1}{n} \end{array}$$ (12)
**Step 5:** Perform the following calculation:
  $$\begin{array}{c} \mu ={\left(L\left(g^{\lambda }\;\text{mod}\;n^{2}\right)\right)}^{-1}\;\text{mod}\;n \end{array}$$ (13)
  Where mod refers to modulo operation. Subsequently, the public key is derived as (*n*, *g*) and the private key as (λ, *μ*).
2) Encryption
**Step 1:** Input the plaintext message *m* to be encrypted, where *m* satisfies 0 < *m* < *n*.
**Step 2:** Choose a random number *a* such that 0 ≤ *a* < *n*, and ensure that $a\in Z_{n}^{*}$.
**Step 3:** Calculate the ciphertext *c* using $g^{m}a^{n}\;\text{mod}\;n^{2}$.
3) Decryption
**Step 1:** Input the ciphertext *c*, ensuring that $c\in Z_{n}^{*}$.
**Step 2:** Calculate the plaintext message *m* using the formula $L(c^{\lambda }\;\text{mod}\;n^{2})\cdot \mu \;\text{mod}\;n$.
  4) Homomorphic Encryption Calculation
  **Homomorphic Addition Calculation:** For ciphertexts *c*_1_ and *c*_2_, the ciphertext *c* is computed using *c*_1_ × *c*_2_.
  **Homomorphic Scalar Multiplication Calculation:** For a ciphertext *c*_1_ and a scalar *b*, the ciphertext *c* is computed as $c=c_{1}^{b}\;\text{mod}\;n^{2}$.

## 3 NIDS-FGPA model building

### 3.1 Model and algorithm flow

**Fig 3:**
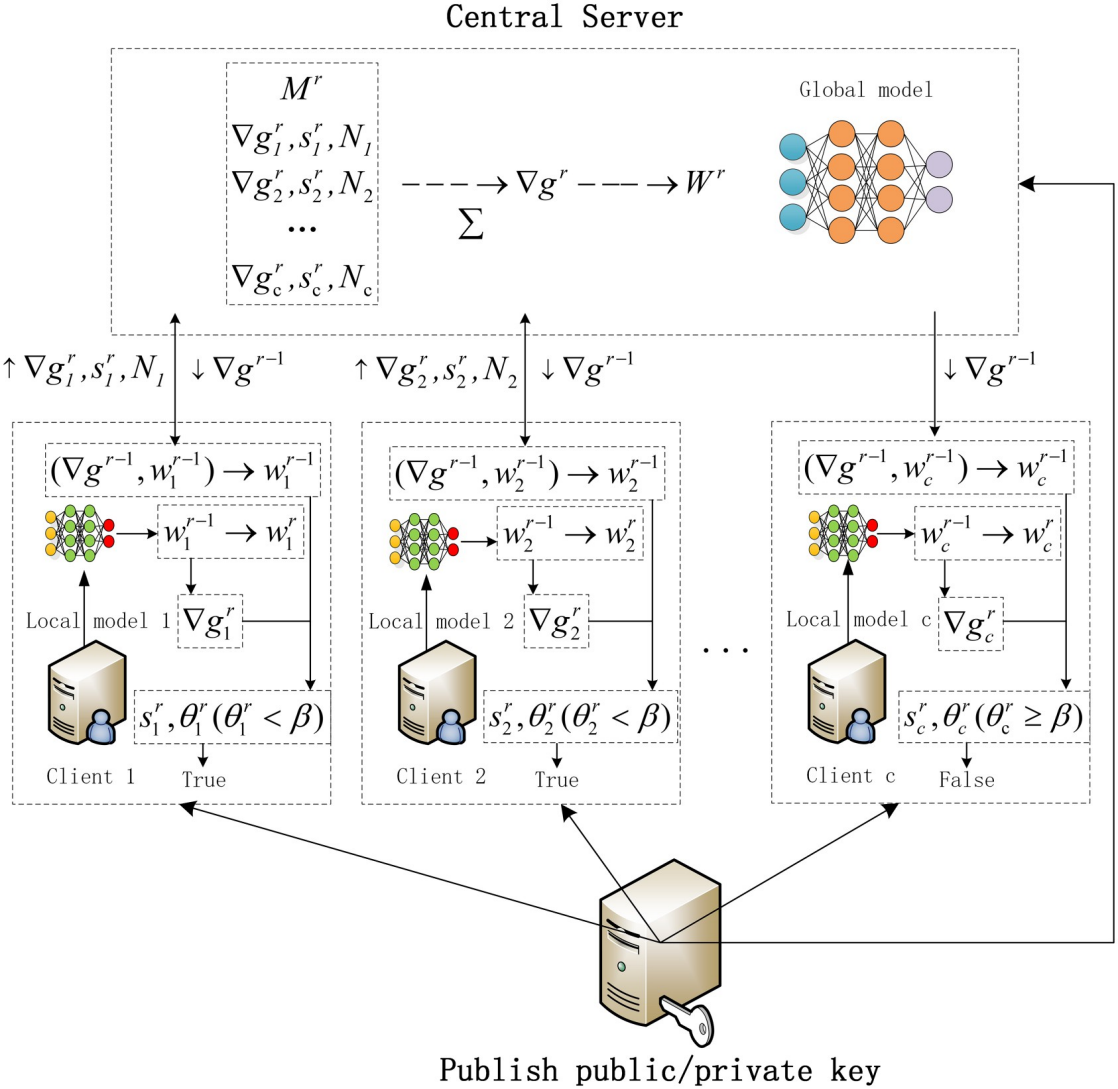
Overall model framework.

The goal of this study is to address common challenges encountered in IIoT, including data feature missingness, incomplete labeling, parameter leakage, and communication overhead. To achieve this objective, we propose the NIDS-FGPA algorithm. In this chapter, we first introduce the dataset used in this paper and describe the steps of data preprocessing and conversion to grayscale images, overcoming the limitations of feature missingness in handling network traffic data by the model. Next, we discuss the internal establishment process of the NIDS-FGPA model, beginning with the introduction of the two-dimensional 2DCNN-BIGRU training model employed at the client side to enhance the model’s ability to detect complex network attacks. Subsequently, we introduce the specific implementation details of the model aggregation algorithm based on gradient similarity, aiming to reduce communication rounds, accelerate model convergence, and minimize communication overhead. Finally, we describe the implementation details of the encryption algorithm, Pallier homomorphic encryption technique, used during the aggregation process to ensure data security and privacy during training. The overall model structure is depicted in Fig 3.

### 3.2 Data preprocessing

In this study, we utilize two widely recognized datasets, namely Edge-IIoTset and CIC IoT 2023, to evaluate the performance of the NIDS-FGPA model in the IIoT environment.

Firstly, the Edge-IIoTset dataset is specifically designed for IIoT intrusion detection, containing a large number of records and multiple features. This dataset covers multiple types of attacks and normal traffic, providing simulated real-world data crucial for evaluating intrusion detection systems [29]. It includes data from diverse IoT devices, such as low-cost digital sensors, ultrasonic sensors, water level sensors, pH sensors, soil moisture sensors, heart rate sensors, and flame sensors, representing different industrial application scenarios.

Secondly, the CIC IoT 2023 dataset offers similarly rich data for evaluating the performance of the NIDS-FGPA model in the IIoT environment. This dataset contains data collected from 33 attacks executed in a topology of 105 IoT devices. These data not only cover multiple attack scenarios but also reflect complex and dynamic network environments, allowing us to test the model’s performance in more challenging settings and further validate its effectiveness in real-world scenarios [30].

By utilizing these two datasets, we can comprehensively assess the effectiveness and reliability of the NIDS-FGPA model. The Edge-IIoTset dataset provides a broad spectrum of normal and attack traffic from multiple IoT devices, while the CIC IoT Dataset 2023 offers detailed data on various attack scenarios, allowing us to rigorously test and validate the model under different conditions [31]. Detailed descriptions of the datasets are available in Table 2.

**Table 2:** Data set classification.

Classification		
Dataset	Traffic Category	Label Number
Edge-IIoTset	Normal	0
	DDoS	1
	Injection	2
	MITM	3
	Malware	4
	Scanning	5
CIC IoT 2023	Benign	0
	DoS	1
	DDoS	2
	Mirai	3
	Spoofing	4
	Recon	5
	Web	6
	BruteForce	7

During the data preparation phase, the Edge-IIoTset, CIC-IDS2017, and CIC IoT 2023 datasets underwent data cleansing, which involved removing rows containing missing values, infinite values (both positive and negative), and eliminating duplicate rows to ensure data quality [32]. Subsequently, the data was scaled using the min-max normalization method to linearly scale the data into a specified range, typically between 0 and 1. The use of min-max normalization helps eliminate differences in the numerical ranges between different features, ensuring uniform standardization of the data. The purpose of this step is to ensure that the data has a consistent numerical range before being transformed into grayscale images, preparing it for subsequent image processing steps. The mathematical expression is as follows:

$$\begin{array}{c} scaled_{X}=\frac{X-min(X)}{max(X)-min(X)} \end{array}$$ (14)

Where *X* represents the original data, *scaled*_*X*_ represents the data after min-max normalization, and *min*(*X*) and *max*(*X*) are the minimum and maximum values of the data, respectively.

Compared to traditional detection methods based on raw datasets, our approach requires less prior knowledge when transforming data into images and achieves better detection accuracy. However, different image transformation methods may result in varying degrees of information distortion, thus affecting the accuracy and efficiency of detection. Mainstream image transformation methods have certain shortcomings that could lead to distorted information in the transformed images, making the selection of a suitable transformation method crucial for detection accuracy and efficiency.

Taking the Edge-IIoTset dataset as an example, each flow record contains 56 valid features, with the 56th feature being the label representing 6 different types of flow. To perform data visualization, we first ensure the consistency of data ranges through normalization. The visualization of the data includes the following three steps:

**Step 1:** Convert the 55 features of the dataset into vectors represented by binary digits. There are two methods to accomplish this step:
  - The first method is to convert the letters into ASCII codes and sum them up, then proportionally convert them into a grayscale image, resulting in a 55-dimensional dataset.
  - The second method involves using one-hot encoding to transform the data. This approach may result in some information loss, as the maximum value in the time feature reaches 13000, far exceeding 255, leading to distortion of proportions and feature loss, thus having certain limitations.
**Step 2:** Pad the transformed 55-dimensional data with zeros to obtain a 64-dimensional image, then convert it into an 8x8 matrix.
**Step 3:** Map the numerical values in the matrix to grayscale pixel values, effectively converting it into a grayscale image, and label the image data for different categories.

**Fig 4:**
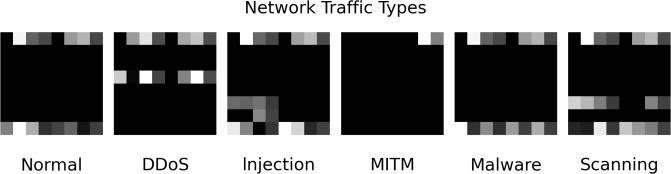
Transformed grayscale image.

This method effectively addresses the issue of feature missing in the model’s processing of network traffic data. The converted image, as shown in Fig 4, where each number represents the grayscale value of the corresponding pixel. The larger the number, the closer the corresponding pixel is to white. By transforming numerical data into images, we can fully leverage the advantages of convolutional neural networks in spatial invariance, thus extracting more effective features [33].

Following this approach, the final size of the grayscale images for the Edge-IIoTset dataset is (8, 8, 1), and for the CIC IoT 2023 dataset is (9, 9, 1).

### 3.3 2DCNN-BIGUR model construction

**Fig 5:**
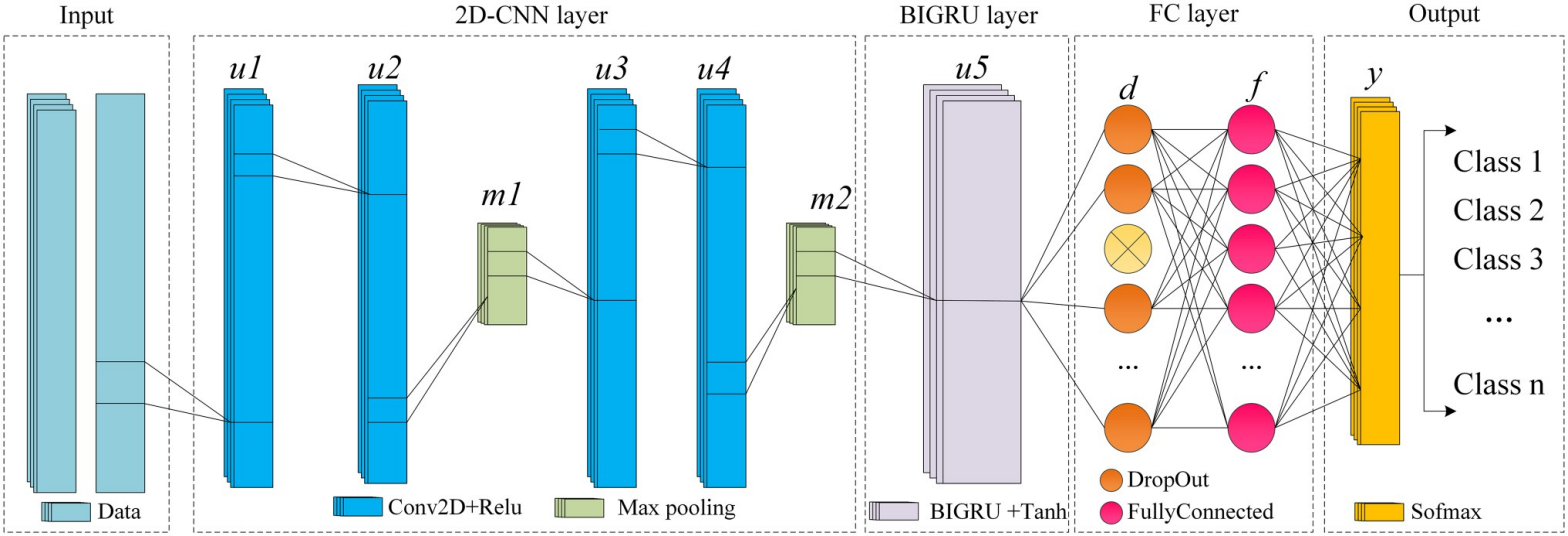
2DCNN-BIGRU framework.

To enhance the model’s capability in detecting complex network attacks, this study combines two deep learning models: 2D-CNN and BIGRU. We designed a 2D-CNN to extract spatial features from network traffic data. Compared to the traditional intrusion detection models using 1D-CNN, the 2D-CNN can more effectively capture spatial features of complex network attack traffic. Additionally, we employ BIGRU to extract temporal features from network traffic data. By designing the 2DCNN-BIGRU deep learning network intrusion detection model (refer to Fig 5), we can thoroughly extract data features, thus enhancing the detection capability [34].

The specific module structure is as follows:

(1)CNN Module:This section comprises four two-dimensional convolutional layers and two max-pooling layers. The preprocessed network traffic data is input as a feature vector *x* to the convolutional units of the CNN module:

$$\begin{array}{c} u_{1}={\text{ConvUnit}}_{1}\left(x\right) \end{array}$$ (15)

$$\begin{array}{c} u_{2}={\text{ConvUnit}}_{2}\left(u_{1}\right) \end{array}$$ (16)

$$\begin{array}{c} m_{1}={\text{MaxPooling}}_{1}\left(u_{2}\right) \end{array}$$ (17)

$$\begin{array}{c} u_{3}={\text{ConvUnit}}_{3}\left(m_{1}\right) \end{array}$$ (18)

$$\begin{array}{c} u_{4}={\text{ConvUnit}}_{4}\left(u_{3}\right) \end{array}$$ (19)

$$\begin{array}{c} m_{2}={\text{MaxPooling}}_{2}\left(u_{4}\right) \end{array}$$ (20)

$$\begin{array}{c} \text{ReLU}(u_{i})=\{\begin{array}{cc} 0 & u_{i}<0\\ u_{i} & u_{i}\geq 0 \end{array} \end{array}$$ (21)

Where ConvUnit_*i*_ represents the *i*-th convolutional unit in the CNN model, *u*_*i*_ denotes the hidden vector. Convolution operations are performed within each convolutional unit, and the ReLU function is used to mitigate model overfitting. Two-dimensional convolution with a window size of 3 and a stride of 1 is adopted in this paper. MaxPooling_*i*_ denotes the *i*-th max-pooling unit, where max-pooling operations are conducted with a pooling window size of 2 and a stride of 1 [35].

(2)BIGRU Module:The data outputted from the CNN module is fed into the BIGRU model to extract temporal features between traffic data. It primarily controls the transmission state through gate control status, remembering the temporal relationships of features [36], and forgetting unimportant information.

$$\begin{array}{c} u_{5}=BIGRU\left(m_{2}\right) \end{array}$$ (22)

Here, *m*_2_ represents the feature data after dimension reduction by the CNN module. BIGRU treats *m*_2_ as a multivariate time series input with a single time step for feature learning. Ultimately, the output of BIGRU, *u*_5_, is obtained.

(3)Fully Connected Module:The output from the previous module is fed as input into the fully connected module, where it undergoes Dropout, fully connected operation, and Softmax operation before outputting the network attack classification result of the model [37]. The corresponding equations are (15) to (17).

$$\begin{array}{c} d=\text{DropOut}(u_{5}) \end{array}$$ (23)

$$\begin{array}{c} f=\text{FullyConnected}(d) \end{array}$$ (24)

$$\begin{array}{c} \tilde{y}=Softmax(f) \end{array}$$ (25)

*u*_5_ serves as the input to the fully connected layer, *f* represents the output after the fully connected operation, *d* denotes the output of the Dropout operation, and *y* is the final output of the classification result. Dropout allows for randomly discarding weight parameters according to a specified ratio to prevent overfitting of the model. $\tilde{y}$ represents the ultimate classification result.

(4)Model Training:Cross-entropy function is used as the loss function in this paper, defined as Eq (18):

$$\begin{array}{c} L=-\sum_{i=1}^{T}y_{i}\;\text{log}\left(\tilde{y_{i}}\right) \end{array}$$ (26)

*T* represents the total number of attack categories, $\tilde{y_{i}}$ represents the model detection label corresponding to the *i*-th category. *y*_*i*_ represents the true label corresponding to the *i*-th category.

### 3.4 Model aggregation algorithm flow for gradient similarity

Specifically, the workflow of one round of FL training using the GSA algorithm is detailed as follows:

**Step 1,Initialization:** The central server first establishes a global CNN-BIRGU deep learning model *G*^0^ and defines the model structure of each layer as well as specific model training parameters (such as the number of local iteration rounds *E*, learning rate *ρ*, batch size *B*, similarity threshold *β*, etc.). Then, the model parameters *W*^0^ and the communication status of all clients are initialized to *true* (i.e., communication is allowed). Finally, the central server sends *G*^0^ and the structural parameters to all participating clients through the communication network.
**Step 2, Local Model Update and Training:** During the first round of communication, upon receiving the training parameters and the global model *G*^0^, each client establishes a local model $L_{0}^{c}$ based on *G*^0^ and sets the training parameters for the local model. Then, the model update process is initiated based on the model parameters *W*^0^ of *G*^0^. During non-first-round communications, the client receives the updated gradient $▽g^{r-1}$ of the parameters from the previous round’s global model *G*^*r*−1^. The specific update process is as follows:
  1. During non-first-round communications, the parameters *W*^*r*−1^ of the previous round’s global model are calculated using the received $▽g^{r-1}$ and the parameters *W*^*r*−2^ of the second-to-last round’s global model (see formula (7)). These calculated parameters are then used to replace the local model parameters $W_{c}^{r-1}$ of the client for the previous round. The client status is set to *True* (the client status applies only to the gradient upload and aggregation phases, not the distribution phase). During the first round of communication, no calculation is required, and the received *W*^0^ is directly used to replace $W_{c}^{r-1}$.
  2. Each client utilizes its local data resources *D*_*c*_ and training parameters to perform gradient descent training on the local deep learning model (before training, the model parameters are $W_{c}^{r-1}$). After the training is completed, the client obtains the local model parameters $W_{c}^{r}$ for the current round as well as the local gradient updates $▽g_{c}^{r}$ (based on formula (7)).
**Step 3: Local Model Selection and Upload:** In this stage, we need to filter out some local models that are useless for the global model by using a similarity threshold *β*. The specific steps are as follows:
  1. After each round of local training, the client calculates the cosine similarity $s_{c}^{r}$ and the gradient vector angle $\theta_{c}^{r}$ based on the local gradient update vector $▽g_{c}^{r}$ and the global gradient update vector $▽g^{r-1}$ (see formulas (8) and (9)). The gradient update vectors represent the directions between the two vectors, and a larger angle indicates a greater inconsistency in direction.
  2. We set the similarity threshold *β* to $\frac{\pi }{2}$. When the vector angle is greater than or equal to $\frac{\pi }{2}$, it indicates that the local update and the global update form a right or obtuse angle, meaning that their update directions are opposite. Uploading these local updates to the central server for weighted aggregation would contribute negatively to the global update. In such cases, we set the client status to *False* to pause communication with the central server.
  3. When the angle is less than $\frac{\pi }{2}$, the local parameter set $p_{c}^{r}=\left(N_{c},▽g_{c}^{r},s_{c}^{r}\right)$ is packaged and uploaded to the central server.
  4. Stop the client update program.
**Step 4, Central Server Dynamic Weighted Aggregation of Model Gradients:** After receiving the parameters sent by clients with the status set to *True*, the central server adds the corresponding client information to the set $M^{r}=\left\{p_{c}^{r}|c=True\right\}$. Subsequently, the gradient updates from the clients in *M*^*r*^ are aggregated. The specific steps are as follows:
  1. First, calculate the sample contribution rate $\mu_{c}^{r}$ based on the sample size of these clients.
    $$\begin{array}{c} \mu_{c}^{r}=\frac{N_{c}}{\sum_{c\in M^{r}}N_{c}} \end{array}$$ (27)
  2. Calculate the similarity $s_{c}^{r}$ using formula (8), and combine it with the *Softmax* function to compute the contribution rate $\lambda_{c}^{r}$ of the local gradient updates from these clients in round *r* (see formula (28)). Using the exponential form of the *Softmax* function can further exaggerate the difference between values, resulting in a smaller contribution rate for poorer quality local models and reducing their impact on the global model.
    $$\begin{array}{c} \lambda_{c}^{r}=softmax\left(s_{c}^{r}\right)=\frac{e^{s_{c}^{r}}}{\sum_{c\in M^{r}}\;e^{s_{c}^{r}}} \end{array}$$ (28)
    Additionally, to mitigate the adverse effects of randomness in each round’s $\lambda_{c}^{r}$, we calculate the average contribution rate ${\bar{\lambda }}_{c}^{r}$ over the recent *R*′ rounds.
    $$\begin{array}{c} {\bar{\lambda }}_{c}^{r}=\frac{1}{R^{\prime }}\sum_{i\in R^{\prime }}\lambda_{c}^{i} \end{array}$$ (29)
  3. Based on $\mu_{c}^{r}$ and ${\bar{\lambda }}_{c}^{i}$, the contribution degree of local updates to the global updates is comprehensively calculated (see formula (30)). Subsequently, using this contribution weight $Weight_{c}^{r}$, the local model gradient updates are weighted and merged to compute the global model gradient $▽g^{r}$ for round *r* (see formula (31)). Then, based on the global model parameters from the previous round and in conjunction with formula (7), the current round’s global model parameters *W*^*r*^ are computed and stored on the central server. Finally, the central server distributes $▽g^{r}$ to all clients.
    $$\begin{array}{cc} Weight_{c}^{r} & =\frac{{\bar{\lambda }}_{c}^{r}\times \mu_{c}^{r}}{\sum_{c\in M^{r}}\;{\bar{\lambda }}_{c}^{r}\times \mu_{c}^{r}} \end{array}$$ (30)
    $$\begin{array}{cc} ▽g^{r} & =\sum_{c\in M^{r}}Weight_{c}^{r}\times ▽g_{c}^{r} \end{array}$$ (31)
**Step 5, Stopping the Training:** After repeatedly training multiple times among Step 2, Step 3, and Step 4, the training is stopped when the global model converges or reaches the preset number of rounds *R*. At this point, the global model becomes the final model for the entire system, with its model parameters being *W*^*R*^ [38].

Furthermore, to provide a more visual representation, the pseudocode is presented in Algorithm 1.

**Algorithm 1** Similarity Calculation

1: **Input:** Communication Rounds *R*, Batch Size *B*, Total Number of Clients *C*, Local Iterations *E*, Client Data *D*_*c*_, Learning Rate *ρ*, Loss Function *L*, Similarity Threshold *β*

2: **Output:** Deep Learning Model

3: **Initial:**

4: 1)The central server initializes the global model parameters *W*^0^ and sets the client status to *True*;

5: 2) Central Server distributes *B*, *E*, *β*, *L*, *ρ*, and *W*^0^ to all Clients

6: **For Server:**

7: **for** *r* ≤ *R* **do**

8:  Start the client update process and receive uploaded parameters from multiple clients: $N_{c},▽g_{c}^{r},s_{c}^{r}=ClientUpdate\left(▽g^{r-1}\right)$;

9:  Collect the obtained client parameters into the set *M*^*r*^, indicating that the communication status of all clients within this set is *True* for the *r*-th round;

10:  Calculate the sample size contribution $\mu_{c}^{r}$ for each client in *M*^*r*^;

11:  Calculate the average contribution of the client side in *M* over R` rounds of gradient updates ${\bar{\lambda }}_{c}^{r}$;

12:  Compute the current round of global gradient updates $▽g^{r}$;

13:  Calculate the global model parameters for this round:$w^{r}=w^{r-1}-\rho \times ▽g^{r}$;

14:  Downcast $▽g^{r}$ to all clients;

15: **end for**

16: **for** Clients *c* **do**

17:  $ClientUpdate(▽g^{r-1}):$

18:   Set the client communication state to *Ture*;

19:   Updating the local model parameters of the customer side $W_{c}^{r-1}\leftarrow W^{r-1}=W^{r-2}-\rho \times ▽g^{r-1}$;

20:   Gradient descent is trained to obtain the local model parameter $w_{c}^{r}$ for this round, which is combined with $W_{c}^{r-1}$ to compute the local gradient update $▽g_{c}^{r}$ for this round;

21:   Compute the gradient update similarity $s_{c}^{r}$;

22:   Calculate the similarity angle $\theta_{c}^{r}=\text{arccos}\left(s_{c}^{r}\right)$;

23:  **if** $\theta_{c}^{r}>\pi /2$ **then**

24:   Client communication status is *False*;

25:  **else**

26:   Upload $▽g_{c}^{r}$, *N*_*c*_, and $s_{c}^{r}$ to Central Server

27:  **end if**

28: **end for**

### 3.5 Homomorphic encryption

The basic workflow of a round of FL for Paillier’s homomorphic encryption algorithm is described as follows:

**step 1, Initialization:** The trust authority generates the public key *pu*_*key* = (*n*, *g*) and the corresponding private key *pr*_*key* = (λ, *μ*) according to the Pallier algorithm described in this paper. Then, the trust authority releases *pu*_*key* and distributes *pr*_*key* to all clients as well as the central server.
**step 2, Local Model Update and Training:** After the client receives the encrypted gradient update $▽g^{r-1}$ of the previous round of the global model from the cloud server, each client performs decryption operations on the encrypted gradient update $En▽g^{r}$ using the private key:
  $$\begin{array}{c} \begin{array}{cc} ▽g^{r-1} & =DeParameter(En▽g^{r-1},pr\_key)\\ & =L({\left(En▽g^{r-1}\right)}^{\lambda }\;\text{mod}\;n^{2})\times \mu \;\text{mod}\;n\\ & =\frac{{\left(En▽g^{r-1}\right)}^{\lambda }\;\text{mod}\;n^{2}-1}{n}\times \mu \;\text{mod}\;n \end{array} \end{array}$$ (32)
  Among them, DeParameter refers to the parameter decryption operation, and *L* is the *L* function described in formula (12). After that, the model update program is initiated.
**step 3, Local Model Selection and Encrypted Upload:** Calculate the similarity $s_{c}^{r}$ and perform model selection. Subsequently, encrypt the filtered local model update gradient $▽g_{c}^{r}$ using the public key to obtain $En▽g_{c}^{r}$. The specific encryption calculation is as follows:
  $$\begin{array}{c} \begin{array}{cc} En▽g_{c}^{r} & =EnParameter(▽g_{c}^{r},pu\_key)\\ & =g^{▽g_{c}^{r}}{\left(a_{c}\right)}^{n}\;\text{mod}\;n^{2} \end{array} \end{array}$$ (33)
  Here, *a*_*c*_ is a random number generated by client *c* that satisfies $a_{c}\in Z_{n}^{*}$. *EnParameter* refers to the parameter encryption operation. The set of encrypted parameters, $EnP_{c}^{r}=\left(N_{c},En▽g_{c}^{r},s_{c}^{r}\right)$, is then packaged and uploaded to the central server.
**step 4, Dynamic Weighted Aggregation Encryption Model Gradient of Central Server:** Calculate the contribution of sample quantity $\mu_{c}^{r}$ from clients in *M*^*r*^ and the average contribution rate $\bar{\lambda_{c}^{r}}$ of gradient updates. Consequently, compute the total contribution weight $Weight_{c}^{r}$ for clients, which is a scalar value. Perform homomorphic weighted aggregation operation *Aggregate* on $Weight_{c}^{r}$ and local encrypted gradient updates $En▽g_{c}^{r}$, resulting in the global encrypted gradient update for the *r*-th round, $En▽g^{r}=Aggregate\left(En▽g_{c}^{r},Weight_{c}^{r}\right)$. Specifically, begin with homomorphic scalar multiplication to obtain the intermediate result $En▽g^{r}{`}_{c}$, followed by homomorphic encryption operation on it:
  $$\begin{array}{c} \begin{array}{cc} En▽g^{r}{`}_{c} & ={\left(En▽g_{c}^{r}\right)}^{Weight_{c}^{r}}\;\text{mod}\;n^{2}\\ & ={\left(g^{▽g_{c}^{r}}{\left(a_{c}\right)}^{n}\;\text{mod}\;n^{2}\right)}^{Weight_{c}^{r}}\;\text{mod}\;n^{2}\\ & =g^{▽g_{c}^{r}\times Weight_{c}^{r}}\times {\left(a_{c}\right)}^{n\times Weight_{c}^{r}}\;\text{mod}\;n^{2} \end{array} \end{array}$$ (34)
  $$\begin{array}{c} \begin{array}{cc} & En▽g^{r}=\prod_{c\in M^{r}}En▽g^{r}{`}_{c}\;\;\;\;\;\text{mod}\;n^{2}\\ & =\prod_{c\in M^{r}}\left(g^{▽g_{c}^{r}\times Weight_{c}^{r}}\times {\left(a_{c}\right)}^{n\times Weight_{c}^{r}}\;\;\;\;\;\;\text{mod}\;n^{2}\right)\;\;\;\;\;\;\text{mod}\;n^{2}\\ & =g^{\sum_{c\in m^{r}}▽g_{c}^{r}\times Weight_{c}^{r}}\times \prod_{c\in M^{r}}\left({\left(a_{c}\right)}^{n\times Weight_{c}^{r}}\right)\;\;\;\;\;\;\text{mod}\;n^{2} \end{array} \end{array}$$ (35)

Finally, the encrypted global gradient $En▽g^{r}$ will be distributed to all clients. The central server can also utilize the key to decrypt the encrypted global gradient, calculate the global model parameters for this round, and obtain the global model for this round.

**step 5, Termination of Training:** After multiple iterations of training between steps 2, 3, and 4, the training stops when the global model converges. At this point, the global model becomes the final model of the entire system, with model parameters represented as *W*^*R*^.

Based on the above workflow, this paper presents an algorithm description (see Algorithm 2).

**Algorithm 2** Homomorphic Encryption Training Process

1: **Input:** Communication rounds *R*, batch size *B*, total number of clients *C*, local iterations *E*, client local data resources *D*_*c*_, learning rate *ρ*, loss function *L*.

2: **Output:** Deep Learning Model

3: **Initial:**

4: Perform the same initialization process as Algorithm 1;

5: **Trust institution:**

6: Generate the private key *pr*_*key* and the public key *pu*_*key*;

7: Publish *pr*_*key* and *pu*_*key*;

8: **For Server:**

9: **for** *r* ≤ *R* **do**

10:  Start the client update program, receive encrypted parameters uploaded by multiple clients: $En▽g_{c}^{r}),N_{c},s_{c}^{r}=ClientUpdate\left(En▽g_{c}^{r-1}\right)$;

11:  Calculate the sample size contribution $\mu_{c}^{r}$ and the average contribution rate of gradient updates $\bar{\lambda_{c}^{r}}$ for clients in *M*^*r*^;

12:  Calculate the local update contribution $Weight_{c}^{r}$;

13:  For all local updates in *M*^*r*^, calculate the encrypted global gradient update: $En▽g^{r}=Aggregate\left(En▽g_{c}^{r},Weight_{c}^{r}\right)$;

14:  Distribute $En▽g^{r}$ to all clients;

15: **end for**

16: **for** Clients *c* **do**

17:  Receive *pr*_*key* and *pu*_*key*;

18:  $ClientUpdate(En▽g^{r-1})$:

19:  Decrypt $▽g^{r-1}=DeParameter\left(En▽g^{r-1},pr\_key\right)$;

20:  Update the client’s local model parameters $w_{c}^{r-1}\leftarrow w^{r}=w^{r-1}-\rho \times ▽g^{r-1}$;

21:  After gradient descent training as in Algorithm 1, compute $▽g_{c}^{r}$, $s_{c}^{r}$, $\Theta_{c}^{r}$ and filter models based on $\Theta_{c}^{r}$;

22:  Encrypt the gradients $En▽g_{c}^{r}=EnParameter\left(En▽g_{c}^{r},pu\_key\right)$;

23:  Upload $En▽g_{c}^{r}$,$s_{c}^{r}$, *N*_*c*_ to the central server;

24: **end for**

## 4 Experimental environment setup

### 4.1 Preparation for experiment

In Table 3 below, the experimental environment for this study is depicted.

**Table 3:** Experimental environment.

Module	Parameter
Processor	Intel (R) Core (TM) i7-8750H
Video Card	NVIDIA GTX1050Ti 4G
Main Frequency	2.20GHz
RAM	40.00 GB
Operating System	Windows 10
Experimental Tool	Python 3.7 and TensorFlow-GPU 2.6.0

### 4.2 Parameter setting

To assess the effectiveness of the model proposed in this paper, we designed two different experimental scenarios, corresponding to *C* = 3 and *C* = 6 respectively. The key distinction between these scenarios lies in randomly partitioning the training set into *C* − *C*_2_ parts to simulate the Non-IID characteristics of partial client data, where *C*_2_ parts contain Non-IID data, such as containing only one attack category [39]. To better validate the algorithm’s effectiveness and mitigate the potential excessive influence of Non-IID data on the global model during training, we set $C_{2}<\frac{1}{2}\times C$. Specifically, when *C* = 3, *C*_2_ = 1, and when *C* = 6, *C*_2_ = 2. The specific experimental parameters are detailed in Table 4.

**Table 4:** Experimental parameters.

Experiment Parameter	Parameter Description	Value
*c*	Number of FL clients	3, 6
*R*	FL communication rounds	100
*β*	Gradient update similarity angle threshold	$\frac{\pi }{2}$
*B*	Batch size	640
*Loss*	Loss function	Cross-entropy loss
*optimizer*	Optimization algorithm	Adam
*p*	Learning rate	0.001
*R*′	Number of rounds for calculating average similarity	5

### 4.3 Evaluation metrics

In network intrusion detection, normal network activities are typically treated as negative samples, while malicious attacks are considered positive samples. When assessing model performance, we focus on several evaluation metrics:True Positives (TP): The model correctly classifies malicious attack samples as positive, demonstrating its ability to detect actual attack behaviors. False Positives (FP): Normal network activities incorrectly classified as positive by the model, potentially leading to false alarms. True Negatives (TN): Normal network activities correctly classified as negative, indicating the model’s ability to identify the absence of actual attacks. False Negatives (FN): Malicious attack samples incorrectly classified as negative, resulting in missed detections. These evaluation metrics are summarized in Table 5.

**Table 5:** Confusion matrix.

Confusion Matrix	Predictive Attack	Predictive Normal
**True Attack**	TP	FN
**True Normal**	FP	TN

Accuracy: The proportion of correctly classified samples (both positive and negative) among all samples in the dataset. It is an important indicator of overall model performance.

$$\begin{array}{c} ACC=\frac{TP+TN}{TP+TN+FP+FN} \end{array}$$ (36)

Precision: The proportion of correctly identified positive samples among all samples classified as positive by the model. It reflects the accuracy of intrusion detection results.

$$\begin{array}{c} Pre=\frac{TP}{TP+FP} \end{array}$$ (37)

F1 Score: The harmonic mean of precision and recall. It provides a balanced assessment of the model’s precision and recall.

$$\begin{array}{c} F1=\frac{2\cdot Pre\cdot Rec}{Pre+Rec} \end{array}$$ (38)

Recall: The proportion of actual positive samples correctly identified by the model among all positive samples in the dataset. It reflects the detection rate of intrusions.

$$\begin{array}{c} Rec=\frac{TP}{TP+FN} \end{array}$$ (39)

Communication Overhead:The communication overhead is mainly evaluated by the number of communication rounds and the total number of transmitted bits *S*. Since the model does not improve the parameter distribution phase by the central server, this study only calculates the total number of bits transmitted during the local model parameter upload phase, with the specific formula provided in (40). In the formula, $s\_bit_{c}^{r}$ represents the size of the parameter file uploaded by client *c* in round *r*, measured in bytes.

$$\begin{array}{c} S=\sum_{r=1}^{R}\sum_{c\in M^{r}}s\_bit_{c}^{r} \end{array}$$ (40)

## 5 Result

### 5.1 Analysis of model aggregation algorithms based on gradient similarity

In this section, we conducted experiments on two datasets, Edge-IIoTset and CIC IoT 2023, to validate the effectiveness of the GSA proposed. We selected a client number of C = 3 for both datasets. By comparing the performance of our proposed GSA with two existing methods, Federated Learning with Novel Communication-Efficient Federated Aggregation (FedNova) [40] and Federated Averaging (FedAvg), the experiments aimed to evaluate the effectiveness of our proposed model aggregation algorithm in enhancing model performance. Key performance metrics, including accuracy, recall, and F1 score, were compared. Detailed results are presented in Table 6.

**Table 6:** Evaluation of model aggregation algorithms.

Model	Edge-IIoTset				CIC IoT 2023			
	Acc	Rec	Pre	F1	Acc	Rec	Pre	F1
FedNova	0.926	0.926	0.921	0.916	0.964	0.964	0.963	0.967
FedAvg	0.891	0.891	0.894	0.885	0.934	0.934	0.934	0.934
**GSA**	**0.945**	**0.945**	**0.940**	**0.940**	**0.992**	**0.992**	**0.992**	**0.992**

In particular, on the Edge-IIoTset dataset, the GSA algorithm achieved an accuracy of 0.945, which is significantly higher than the accuracies of 0.926 and 0.891 achieved by the FedNova and FedAvg algorithms, respectively. Similarly, on the CIC IoT 2023 dataset, the GSA algorithm exhibited outstanding performance, with an accuracy of 0.992, far surpassing the accuracies of 0.964 and 0.934 obtained by the FedNova and FedAvg algorithms, respectively.

**Fig 6:**
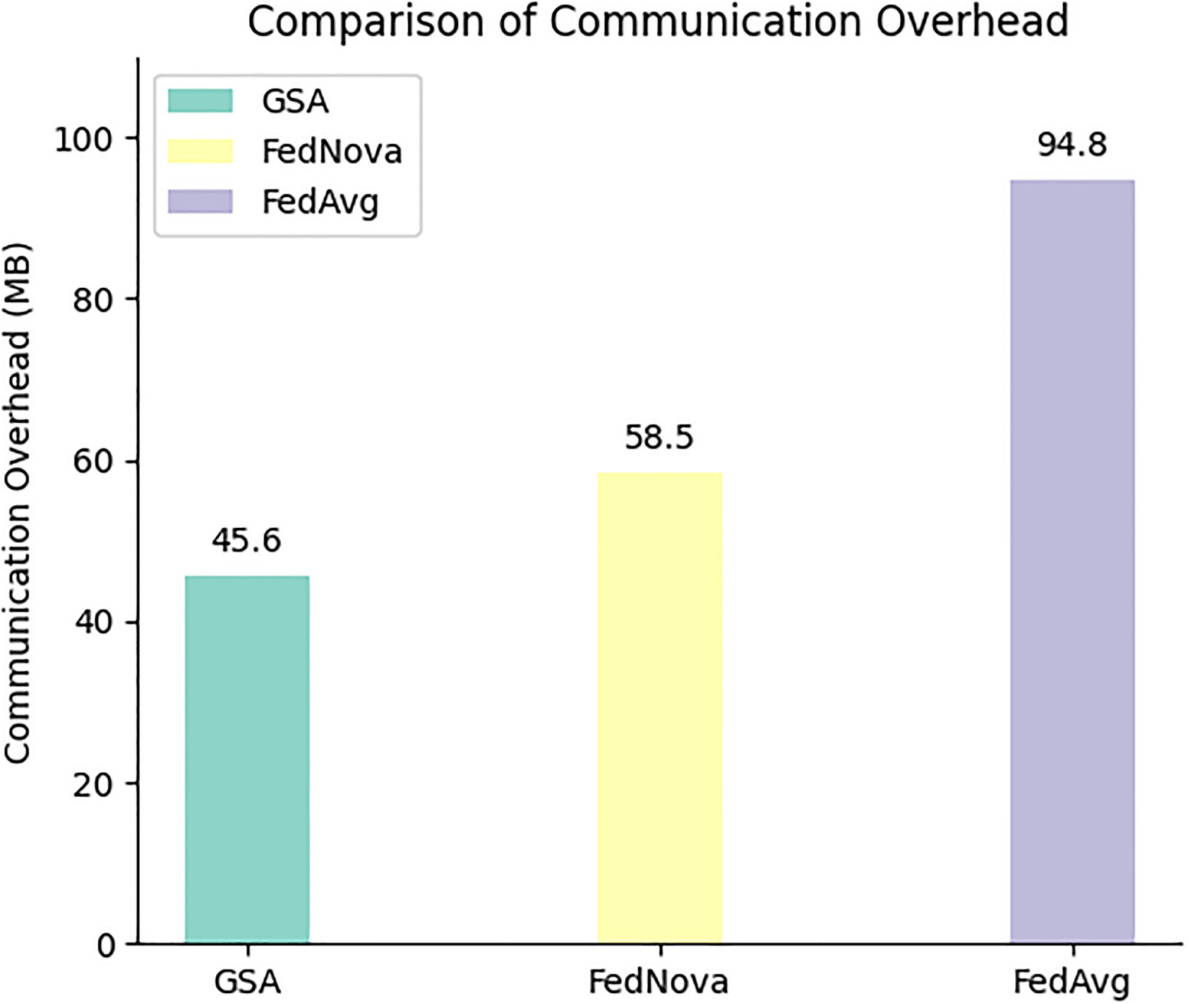
Network resource consumption comparison for three methods in learning process under C = 3 scenario.

To better demonstrate the superiority of the proposed method, this study measured the communication overhead during the learning process for three different methods. The specific results are shown in Fig 6. The experiments were conducted in a scenario with three client instances (*C* = 3).

Overall, compared to the other two methods, GAS reduced communication overhead by 22%-51%. The reason for these observations is that our improved method not only filters client-side models during the model update phase, excluding locally updated models that are irrelevant to global updates, but also calculates the contribution of remaining local updates during the aggregation phase, using it as the basis for dynamic weighted aggregation. This highlights the role of well-performing local updates. In contrast, FedNova and FedAvg have their shortcomings. However, FedNova’s normalized averaging gradient feature leads to slower convergence speeds and increased system costs and complexity when handling large-scale, complex datasets. FedAvg simply averages all local model updates, unable to effectively filter out locally updated models that contribute less or are irrelevant to the global model, potentially resulting in unnecessary gradient update file uploads during communication, increasing communication overhead. Therefore, our improved method is less affected by adverse local model effects and can achieve higher accuracy. This means that the improved method will reduce the number of communication rounds and the frequency of uploading gradient files locally, while also reducing resource consumption generated by direct communication between servers and clients.

### 5.2 Analysis of the Paillier homomorphic encryption algorithm

**Fig 7:**
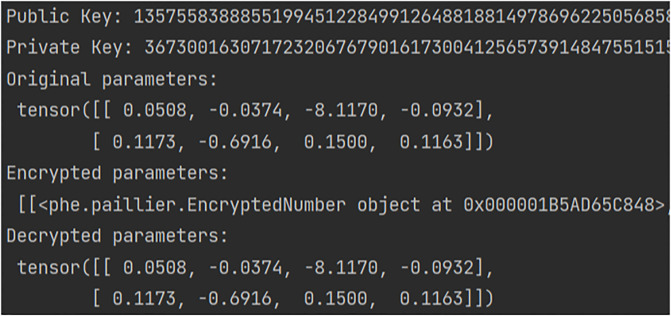
Paillier homomorphic encryption and decryption.

Based on the experimental results, it is observed that the gradient parameters of the model itself are enormous. Therefore, in this study, only a subset of gradient vector data was selected for homomorphic encryption experiments, as illustrated in Fig 7. The encryption and decryption processes of a portion of gradient vectors from a client are demonstrated. The experimental findings indicate that the ciphertext resulting from arithmetic operations (addition and scalar multiplication) on the encrypted gradient vectors yields decrypted results consistent with those obtained from corresponding operations on plaintext. This confirms the effectiveness of our optimization.

Following that, we generated public and private keys of varying sizes corresponding to key lengths of 256 bits, 512 bits, 1204 bits, and 2048 bits, respectively. Subsequently, we conducted experiments on the intrusion detection model we designed. We collected data on the time and ciphertext size consumed during encryption, decryption, and ciphertext aggregation stages. Additionally, the size of our model’s gradient update parameters, when in plantext, was 0.67MB. The results are presented in Table 7.

**Table 7:** Comparison of different key lengths.

Key size	Encryption duration (seconds)	Decryption duration (seconds)	Encrypted aggregation duration (seconds)	Ciphertext size (MB)
256*bit*	4.87	1.36	5.86	5.67
512*bit*	15.98	5.12	11.56	12.6
1024*bit*	89.95	23.96	36.87	24.3
2048*bit*	587.05	130.23	213.89	53.7

**Fig 8:**
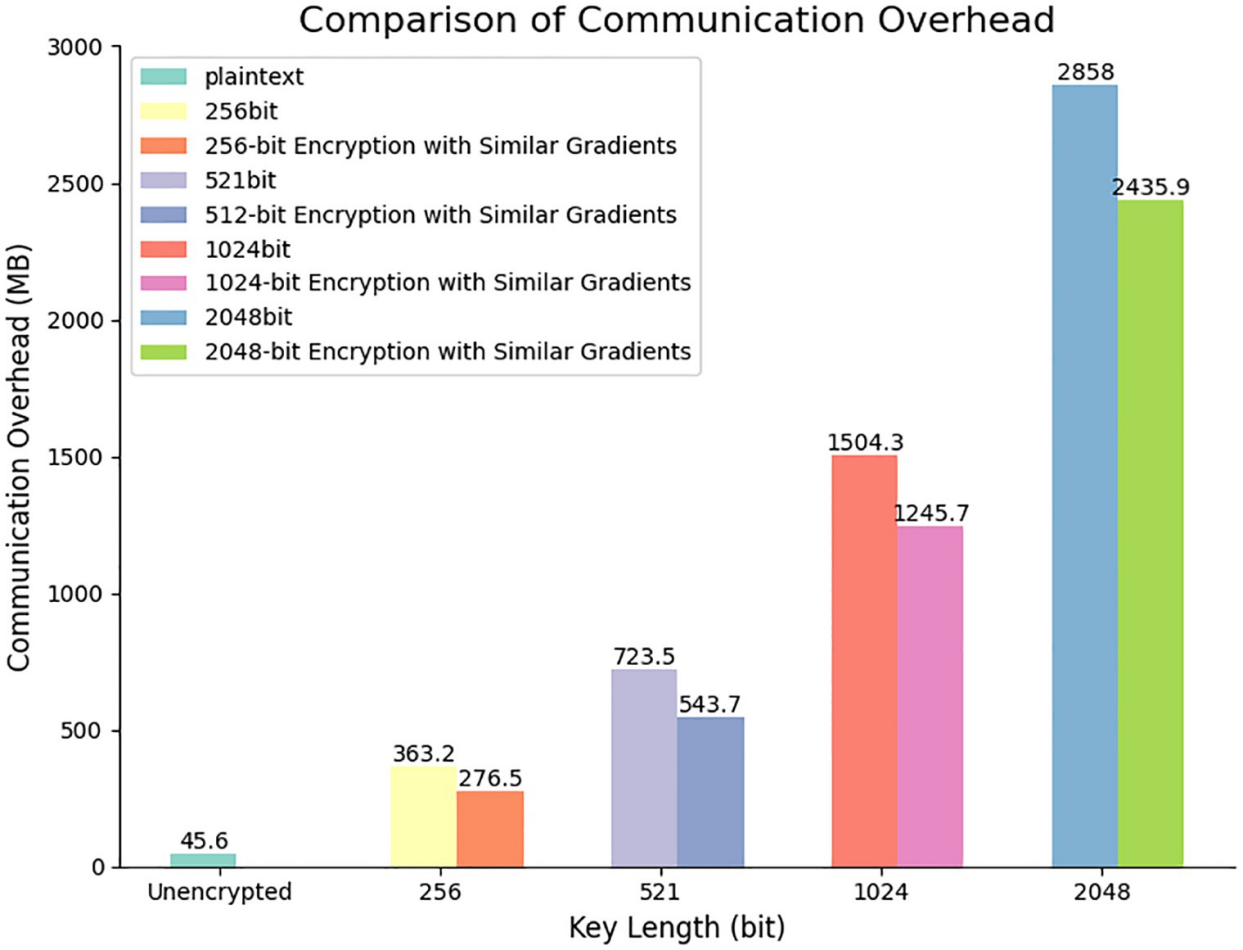
Communication overhead under different key lengths.

As the length of the encryption key increases, so does the time required for encryption, decryption, and encrypted aggregation. Furthermore, the size of gradient update parameters, initially 0.67MB in plaintext, expands to 5.67MB, 12.6MB, 24.3MB, and 53.7MB after encryption using keys of varying lengths. The ciphertext size is several times larger than the plaintext, indicating that ciphertext transmission entails greater communication overhead compared to plaintext transmission. To thoroughly assess the impact of the Paillier homomorphic encryption algorithm on model performance, we conducted experiments with three client instances (*C* = 3). In this experiment, we compared the communication overhead under three conditions: plaintext transmission using the gradient similarity model aggregation algorithm, encrypted transmission using the gradient similarity model aggregation algorithm, and encrypted transmission without utilizing the gradient similarity model aggregation algorithm. The detailed outcomes are illustrated in Fig 8.

After encryption, it can be observed that the communication overhead increases significantly. Taking the example of a 256-bit key length, the communication overhead has already reached 363.2MB, which is much higher than the 45.6MB of plaintext transmission.

When comparing the usage of the GSA with not using this algorithm, the communication overhead for 256-bit encryption is 276.5MB and 363.2MB respectively, with the former being slightly lower than the latter.

As the key length increases, the communication overhead gradually increases. However, when using the gradient similarity model aggregation algorithm, the communication overhead generated by keys of the same length is usually lower than when not using this algorithm.

Therefore, in practical applications, it is necessary to consider factors such as security, communication overhead, and latency comprehensively to select an appropriate key length. In this study, a key length of 1024 bits was chosen, which has higher security compared to 256 bits and 512 bits. Compared to 2048 bits, this choice reduces nearly half of the communication overhead and also decreases encryption time, decryption time, and aggregation time by factors of 5, 6, and 5 respectively.

### 5.3 Simulated experiment

In this section, we conducted experimental analyses on two datasets: Edge-IIoTset and CIC-IoT 2023. Our evaluation primarily focuses on key metrics, including training accuracy, training loss, and classification reports, to assess the performance of the NIDS-FGPA model on these datasets.

**Fig 9:**
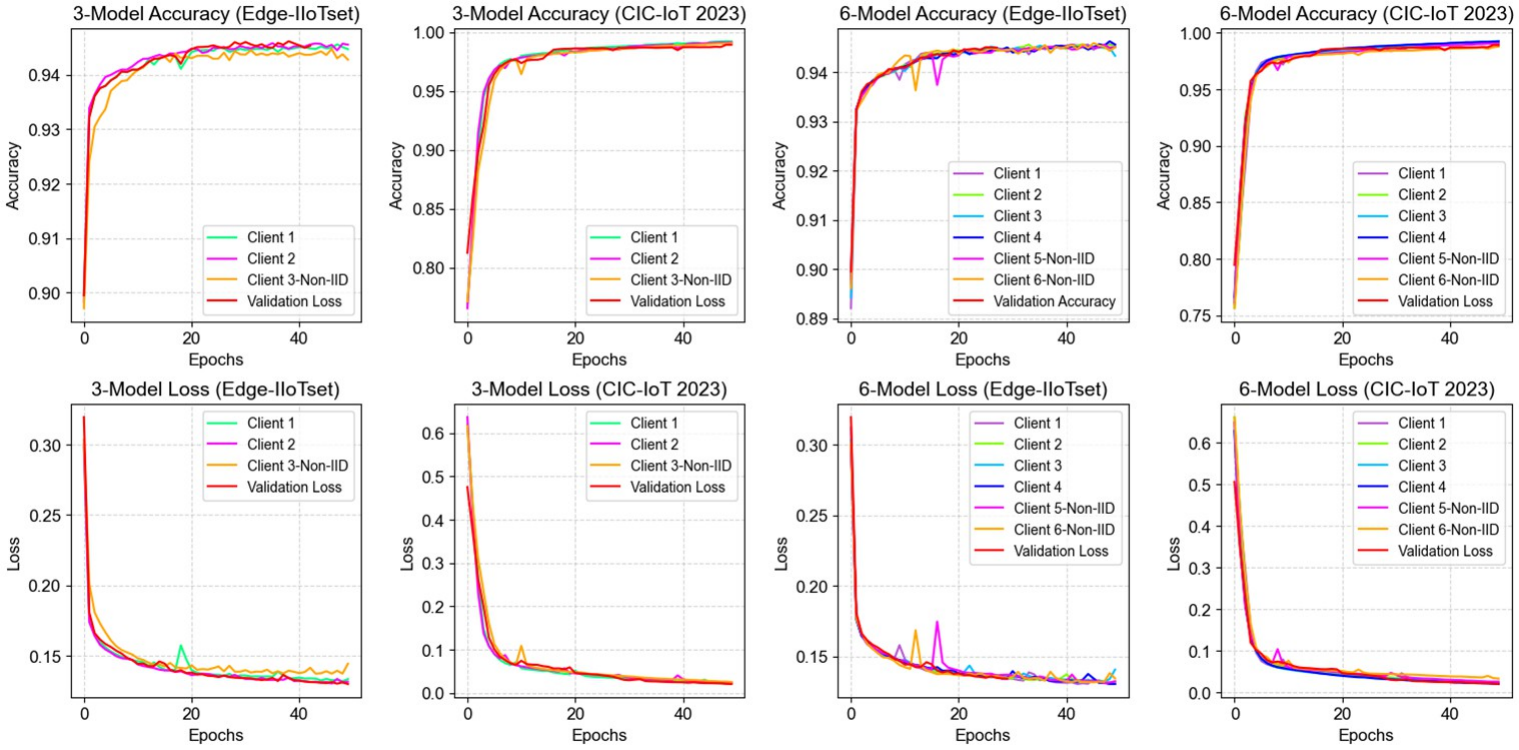
Accuracy and loss variation of the NIDS-FGPA model.

In the cases of *C* = 3 and *C* = 6, we investigated the performance of the NIDS-FGPA model during the training process. The training accuracy represents the proportion of correct predictions made by the model on the training set, while the training loss reflects the level of errors during the training phase. Validation accuracy and validation loss measure the predictive accuracy and error level of the model on the validation set. These metrics help evaluate the learning capability and fitting of the model during the training process and are crucial for further performance analysis. As shown in Fig 9.

In the illustrated results, we observe a gradual convergence of the NIDS-FGPA model during the training process for *C* = 3 and *C* = 6 on the CIC-IoT2023 and Edge-IIoTset datasets. As the number of training epochs increases, the model’s accuracy and loss stabilize gradually. This indicates that the model progressively converges during the training process and is able to achieve a relatively stable performance state.

In the case of *C* = 3, considering the Edge-IIoTset dataset, after 50 iterations, the model achieves training accuracies of 0.947 and 0.945 for Client 1 and Client 2, respectively, with corresponding training losses of 0.133 and 0.131. However, when faced with non-identically distributed data from Client 3, the model’s performance exhibits bias, with the training accuracy dropping to 0.942 and the training loss increasing to 0.144. This performance disparity could stem from the unique data distribution of Client 3, making it challenging for the model to capture its distinct data characteristics effectively.

On the CIC-IoT2023 dataset, after 50 iterations, the model achieves training accuracies of 0.991 and 0.992 for Client 1 and Client 2, respectively, with corresponding training losses of 0.021 and 0.023. However, in the case of Client 3, the model’s performance exhibits bias, with a training accuracy of 0.990 and a training loss of 0.025. This performance disparity could arise from significant differences in the data characteristics of Client 3 compared to the other clients, leading to performance bias.

On the overall validation set, the model demonstrates robust performance. On the Edge-IIoTset and CIC-IoT2023 datasets, the validation accuracies are 0.945 and 0.992, respectively, with validation losses of 0.129 and 0.020. These results indicate that the model exhibits good generalization capability under the setting of *C* = 3.

In the case of *C* = 6, considering the Edge-IIoTset dataset, after 50 iterations, the model achieves training accuracies of 0.945, 0.945, 0.945, and 0.945 for Clients 1, 2, 3, and 4, respectively, with corresponding training losses of 0.130, 0.132, 0.130, and 0.132. However, when faced with non-identically distributed data from Clients 5 and 6, the model’s performance exhibits bias, with training accuracies of 0.943 and 0.945 and training losses of 0.140 and 0.134, respectively. This performance disparity could stem from the uneven data distribution of Clients 5 and 6 compared to the other clients, leading to insufficiently learned features on these clients, thereby affecting performance.

On the CIC-IoT2023 dataset, after 50 iterations, the model achieves training accuracies of 0.992 for Clients 1, 2, 3, and 4, with corresponding training losses of 0.020, 0.021, 0.023, and 0.021, respectively. However, in the cases of Clients 5 and 6, the model’s performance decreases, with training accuracies of 0.987 and 0.990 and training losses of 0.033 and 0.026, respectively. This further underscores the challenges the model faces in handling different data distributions, indicating the need for further optimization and adjustments to enhance its generalization capability.

On the overall validation set, the model demonstrates robust performance. On the Edge-IIoTset and CIC-IoT2023 datasets, the validation accuracies are 0.945 and 0.992, respectively, with validation losses of 0.130 and 0.020. These results indicate excellent performance under the setting of *C* = 6.

**Fig 10:**
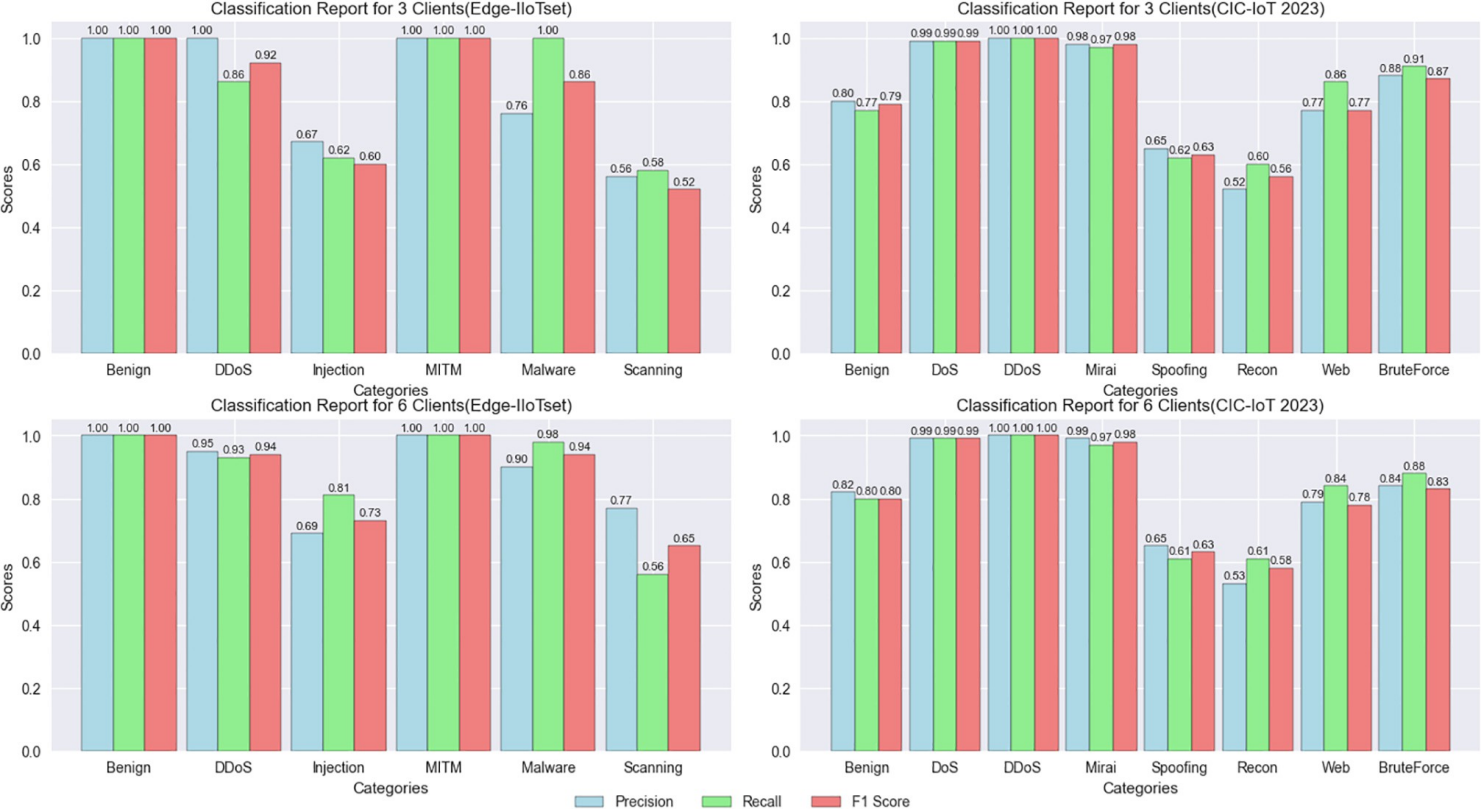
Classification report for the nids-fgpa model.

To comprehensively assess the performance of the NIDS-FGPA model under both *C* = 3 and *C* = 6 scenarios, we conducted a thorough analysis of its performance on the Edge-IIoTset and CIC-IoT 2023 datasets. We provided detailed presentations of key evaluation metrics, including recall, precision, and F1 scores, and delved into an in-depth analysis of the NIDS-FGPA model’s performance on each class. These metrics play a crucial role in comprehensively evaluating the overall performance of the model across different datasets. For specific details regarding the classification report, please refer to Fig 10.

Regarding the Edge-IIoTset and CIC-IoT 2023 datasets, we evaluated the performance of the NIDS-FGPA model under different numbers of clients and obtained precision, recall, and F1 scores. Here is our detailed analysis of the results:

For the Edge-IIoTset dataset, we observed that with three clients, the model showed significant precision and recall in most classes (such as ‘Benign’ and ‘MITM’). However, in classes like ‘Injection’ and ‘Scanning’, the model’s recall was relatively low, suggesting a need for further improvement. Conversely, with six clients, the model’s performance improved across all classes, particularly in classes like ‘Injection’ and ‘Malware’, where both precision and recall showed significant enhancements.

On the CIC-IoT 2023 dataset, with three clients, the model exhibited high precision and recall in most classes (such as ‘DoS’ and ‘DDoS’). However, in classes like ‘Spoofing’ and ‘Recon’, the model’s recall was slightly lower, indicating some overlooked instances. As the number of clients increased to six, the model’s performance remained stable across most classes, with slight improvements in precision and recall in certain classes (such as ‘BruteForce’).

These results suggest that increasing the number of clients can significantly enhance the performance of the NIDS-FGPA model. However, further improvement is needed for certain attack classes (such as ‘Injection’, ‘Malware’, ‘Spoofing’, ‘Recon’). This necessitates more meticulous adjustments and optimizations tailored to the data of specific classes.

Based on the comprehensive analysis above, the GSA in this paper plays a pivotal role in addressing models with Non-IID data. Initially, during the model updating phase, it effectively mitigates the adverse impact of subpar local models on overall performance, particularly when confronted with Non-IID data. Subsequently, in the aggregation phase, dynamic weighted aggregation is conducted based on the contributions of local updates, thereby accentuating the influence of superior-performing local updates on the overall model. This approach adeptly mitigates the ramifications of Non-IID data’s local models on overall performance, consequently reducing the overall communication overhead of the model and enhancing aggregation efficiency.

By leveraging techniques to convert textual data into grayscale image data, the challenge posed by missing data features in traditional deep learning models is successfully surmounted. Furthermore, the application of 2D-CNN amplifies the model’s capability to detect intricate network attacks, particularly with regard to complex local models such as Non-IID data, exhibiting heightened precision and thereby augmenting overall accuracy. Additionally, the incorporation of the Paillier homomorphic encryption algorithm safeguards client data privacy by encrypting gradient vector data, effectively forestalling the leakage of sensitive information.

The application of this technology enables clients to securely participate in the model aggregation process while ensuring the privacy of their data, thereby furnishing crucial safeguards for the establishment of a reliable federated learning environment. Collectively, these methodologies foster the enhancement of model performance and the practicability of applications.

### 5.4 Contrast experiment

To assess the feasibility of the NIDS-FGPA model, we utilized the methods previously proposed on the Edge-IIoTset and CIC IoT 2023 datasets to compare its performance in terms of ACC, Rec, Pre, and F1 score with those of previously proposed models. The comparative results are presented in Table 8.

**Table 8:** Multi-classification performance comparison.

Model	Edge-IIoTset				CIC IoT 2023			
	Acc	Rec	Pre	F1	Acc	Rec	Pre	F1
SVM [41]	0.856	0.890	0.89	0.88	/	/	/	/
KNN [41]	0.839	0.845	0.875	0.851	/	/	/	/
DNN [41]	**0.960**	0.841	0.918	0.86	/	/	/	/
LR [42]	/	/	/	/	0.831	0.696	0.512	0.539
Adaboost [42]	/	/	/	/	0.351	0.487	0.464	0.368
DNN [42]	/	/	/	/	0.991	0.906	0.679	0.697
BiLSTM [43]	/	/	/	/	0.930	0.930	0.913	0.917
DL-BiLSTM [43]	/	/	/	/	0.931	0.931	0.918	0.919
**NIDS-FGPA**	0.945	**0.945**	**0.940**	**0.940**	**0.992**	**0.992**	**0.992**	**0.992**

From the results in Table 8, it can be observed that the NIDS-FGPA model performs exceptionally well on these two datasets.

On the Edge-IIoTset dataset, the NIDS-FGPA model achieves an accuracy of 0.945. Compared to other models, it demonstrates the best performance in terms of recall, precision, and F1 score, with values of 0.945, 0.940, and 0.940, respectively. Although there is room for improvement in accuracy, its overall performance remains remarkable.

Similarly, on the CIC IoT 2023 dataset, the NIDS-FGPA model exhibits outstanding performance with an accuracy of 0.992. Compared to other models, it also achieves the best performance in terms of recall, precision, and F1 score, all with values of 0.992. This further confirms the superior performance of the NIDS-FGPA model on the CIC IoT 2023 dataset.

In summary, the NIDS-FGPA model demonstrates exceptional performance on both the Edge-IIoTset and CIC IoT 2023 datasets, with high accuracy and other evaluation metrics, indicating its excellent performance in classification tasks. This suggests its potential application prospects in the field of network intrusion detection.

## 6 Conclusion

This study conducted experiments on the Edge-IIoTset and CIC IoT 2023 datasets to evaluate the performance of the proposed NIDS-FGPA model in the field of network intrusion detection. The experimental results demonstrate that the NIDS-FGPA model performs excellently on both datasets, significantly outperforming existing methods such as BiLSTM, KNN, and DL-BiLSTM.

The introduced GSA algorithm plays a crucial role in reducing communication overhead. By selecting client models during the model update phase, it effectively mitigates the negative impact of local models on overall performance. Furthermore, in the aggregation phase, dynamic weighted aggregation based on the contributions of local updates highlights the importance of well-trained local updates to the overall model. This approach effectively mitigates the impact of locally non-iid data on overall performance, improving overall accuracy.

Simultaneously, we successfully addressed the challenge of incomplete features in traditional deep learning models by converting text data into grayscale image data. The application of 2D-CNN further enhances the model’s ability to detect complex network attacks. Additionally, the introduction of the Paillier homomorphic encryption algorithm helps protect customer data privacy, making the entire model more efficient.

Future research could further improve the GSA algorithm and explore its performance on other domains or different datasets. Overall, this study provides valuable theoretical support and practical experience for the field of network intrusion detection, offering important insights for building a more secure and reliable network environment.
